# Phase 2, single-arm trial to evaluate the effectiveness of darbepoetin alfa for correcting anaemia in patients with myelodysplastic syndromes

**DOI:** 10.1111/j.1365-2141.2008.07181.x

**Published:** 2008-08

**Authors:** Janice Gabrilove, Ronald Paquette, Roger M Lyons, Chaudhry Mushtaq, Mikkael A Sekeres, Dianne Tomita, Lyndah Dreiling

**Affiliations:** 1 Department of Medicine, Mt Sinai School of MedicineNew York, NY; 2 UCLA Oncology CenterLos Angeles, CA; 3 Cancer Care Centers of South Texas/US Oncology ResearchSan Antonio, TX; 4 Department of Medical Oncology, South Carolina Oncology AssociatesColumbia, SC; 5 The Taussig Cancer Center, Cleveland ClinicCleveland, OH; 6 Amgen Inc., Thousand OaksCA, USA

**Keywords:** myelodysplastic syndromes, erythropoiesis-stimulating agent, erythroid response, haemoglobin, transfusion

## Abstract

Patients with myelodysplastic syndromes (MDS) often develop anaemia resulting in frequent transfusions and fatigue. Darbepoetin alfa is an erythropoiesis-stimulating agent (ESA) approved for treating chemotherapy-induced anaemia. This single-arm, phase 2 study examined the efficacy of darbepoetin alfa 500 μg every 3 weeks (Q3W) for treating anaemia in low-risk MDS patients (after 6 weeks, poor responders received darbepoetin alfa 500 μg every 2 weeks). The primary end-point was the incidence of erythroid responses (International Working Group criteria) after 13 weeks of therapy. Secondary end-points included the incidence of erythroid responses at weeks 28 and 55, [or weeks 27 and 53 for dose escalations to every two weeks (Q2W)], and safety parameters. Analyses were stratified by the patient's previous ESA therapy status [ESA-naïve (*n* = 144) vs. prior ESA-treated (*n* = 62)]. After 13 weeks of therapy, 49% of ESA-naïve patients and 26% of prior ESA-treated patients achieved a major erythroid response. After 53/55 weeks, 59% of ESA-naïve patients and 34% of prior ESA-treated patients achieved a major erythroid response; 82% of ESA-naïve patients and 55% of prior ESA-treated patients achieved target haemoglobin of 110 g/l. Thromboembolic or related adverse events occurred in 2% of patients; no pulmonary embolisms were reported. In conclusion, darbepoetin alfa, 500 μg Q3W appeared well tolerated and increased haemoglobin levels in low-risk MDS patients.

The myelodysplastic syndromes (MDS) comprise a haematologically and biologically diverse group of stem cell disorders characterized by accelerated apoptosis and ineffective haematopoiesis that lead to functional haematopoietic failure (Faderl & Kantarjian, 2004; Catenacci & Schiller, 2005; Hofmann & Koeffler, 2005; Kurtin, 2006). Classification systems for distinguishing MDS subtypes associated with specific prognostic significance have been developed and include the French–American–British (FAB) system, the International Prognostic Scoring System (IPSS) and the World Health Organization (WHO) classification system (Bennett *et al*, 1982; Greenberg *et al*, 1997; Vardiman *et al*, 2002). These classification systems are used clinically in MDS patients to predict both survival and the risk of evolving to acute myeloid leukaemia (AML).

Refractory anaemia (RA) resulting from ineffective erythropoiesis is a major cause of morbidity and mortality in patients with low-risk MDS (Bowen & Hellstrom-Lindberg, 2001; Hellstrom-Lindberg *et al*, 2003; Hofmann & Koeffler, 2005; Kurtin, 2006). Inflammatory cytokines, including tumour necrosis factor-alfa, transforming growth factor-beta and interleukin-1 beta have been implicated in the accelerated apoptosis responsible for ineffective haematopoiesis and lineage-specific cytopenias (including anaemia) associated with MDS (Raza *et al*, 1996; Allampallam *et al*, 2002; Faderl & Kantarjian, 2004; Stifter *et al*, 2005; Verma & List, 2005; Sekeres & List, 2006; Tehranchi, 2006). These inflammatory cytokines can also interfere with endogenous erythropoietin (eEPO) production (Jelkmann *et al*, 1994) which may contribute further to the anaemia observed in MDS patients. Though red blood cell (RBC) transfusions are often used to treat anaemia in patients with MDS disorders, transfusions can be complicated by infection, difficult cross match and subsequent iron overload (Gupta *et al*, 1999; Gardin & Fenaux, 2004; Balducci, 2006). As an alternative to transfusions, studies have explored the efficacy of erythropoiesis-stimulating agents (ESAs) [alone and in combination with granulocyte colony-stimulating factor (G-CSF)] to correct MDS-induced anaemia by overcoming the inhibitory effects of pro-apoptotic cytokines, promoting survival of erythroid progenitors and enhancing erythropoiesis (Hellstrom-Lindberg *et al*, 1998, 2003; Hellstrom-Lindberg, 2005; Musto *et al*, 2005; Spiriti *et al*, 2005; Stasi *et al*, 2005; Balleari *et al*, 2006; Mannone *et al*, 2006). A review of published studies of low-risk MDS patients found a statistically significant benefit of growth factor therapy (including ESAs with or without colony stimulating factors) to both overall survival and progression-free survival compared with non-growth factor therapies (including differentiating agents, cytotoxic agents and immunomodulators). This improvement was observed at 6, 12, 18 and 24 months of follow-up and was independent of FAB subtype, baseline transfusion need, IPSS score, previous treatment and time since diagnosis (Golshayan *et al*, 2007). Some studies have also identified baseline classifications in low-risk MDS patients that predicted a favourable haematological response to ESA treatment. These prognostic factors included eEPO concentrations ≤100 mU/ml, two or fewer RBC transfusions in the previous month and no excess blasts and/or hypoplastic bone marrow (Rose *et al*, 1995; Hellstrom-Lindberg *et al*, 2003; Musto *et al*, 2005). In patients with low transfusion needs and low eEPO concentrations, a decision model predicted a good chance of favourable response to ESA treatment with or without G-CSF, and improved overall survival and quality-adjusted life years (Sekeres *et al*, 2007).

Darbepoetin alfa is a novel ESA that can be administered at extended dosing intervals, such as once every 3 weeks (Q3W) (Kotasek *et al*, 2003; Canon *et al*, 2006). This ESA is approved in the United States and Europe for the treatment of anaemia in patients with chronic renal failure and for the treatment of chemotherapy-induced anaemia (CIA) in cancer patients (Vansteenkiste *et al*, 2002; Hedenus *et al*, 2003; Schwartzberg *et al*, 2004; Herrington *et al*, 2005; Robinson & Easthope, 2005; Canon *et al*, 2006; Glaspy *et al*, 2006).

This single-arm phase 2 study evaluated the biological activity and efficacy of darbepoetin alfa in patients with low- or intermediate-1-risk MDS. The primary end-point was the percentage of patients achieving either a major or minor erythroid response (using the International Working Group (IWG) criteria) (Cheson *et al*, 2000) following the initial 13 weeks of darbepoetin alfa therapy. Prospectively defined secondary end-points evaluated the efficacy of darbepoetin alfa at 27/28 weeks and 53/55 weeks on the percentage of patients achieving an erythroid response, the change in haemoglobin concentration from baseline, the incidence of transfusions, the change in patient-reported fatigue, and incidence of adverse events.

## Patients and methods

### Study population

The original protocol approved on 1 March 2004 specified a sample size of 120 patients who had not been previously treated with an ESA. A protocol amendment was approved on 18 May 2005 to allow patients who had previously received an ESA to be enroled (prior ESA therapy had to be stopped for 7 to 30 d before study enrolment). This amendment also increased the total sample size to 200 patients. This study was designed as an estimation study to determine major and minor response rates when darbepoetin alfa was administered to patients with low-risk MDS with and without prior ESA treatment to assist in planning future MDS studies.

Written approval was received from the Institutional Review Board at each study centre. Before study procedures (including screening) were initiated, informed consent was obtained from patients. All screening tests were performed within a maximum of 7 d before enrolment, with eligible patients being enroled within 7 d after completion of screening. An interactive voice-response system was called to obtain a unique identification number for each patient. Patients who terminated prematurely from the study were not replaced.

Patients ≥18 years were eligible for this study if they had a haemoglobin concentration ≤110 g/l, adequate iron stores, Eastern Cooperative Oncology Group status score of 0 to 2, adequate renal and liver function and low-risk MDS (low- or intermediate-1-risk as defined by the IPSS) with a FAB classification of RA, RA with ringed sideroblasts (RARS) or RA with excess blasts (RAEB) (with blasts ≤10%). FAB and IPSS classifications were determined by complete blood cell counts and bone marrow biopsies obtained within 8 weeks before enrolment for ESA-naïve patients or 8 weeks before initiation of ESA therapy for currently treated patients.

Patients were excluded from this study if they had a previous bone marrow or stem cell transplant, history of transfusion-dependent thrombocytopenia, chronic myelomonocytic leukaemia, history of pure red cell aplasia, class II or IV cardiac disease, uncontrolled hypertension, clinically significant systemic infection or chronic inflammatory disease, anaemia related to nutritional deficiencies or other causes (including haemolysis, bleeding, sickle-cell anaemia and renal disease), history of malignancies other than MDS and previous or ongoing treatment with biological response modifiers except for ESAs (which had to be discontinued at least 7 d and not more than 30 d before enrolment) or G-CSF, which could be used for neutropenic fever or infection during the 30 d before study enrolment. Patients were also excluded if they received investigational agents not approved for any indication during the 30 d before enrolment, received ongoing corticosteroid therapy (other than as premedication for transfusions), received methotrexate or MDS-targeted therapy (including chemotherapy, antibody-based cancer treatments, hormonal therapies, interferons and interleukins) within 30 d before screening, received radiotherapy in the year before screening or planned to receive chemotherapy or radiotherapy within 28 weeks after enrolment. Additional reasons for patient exclusion included a positive human immunodeficiency virus test, positive antibody response to an ESA, known failure of ESAs to treat anaemia and known hypersensitivity to darbepoetin alfa or any of its excipients. Patients were also excluded if they were pregnant, breast-feeding or not using adequate contraception.

### Study design

This was a phase 2, stratified, single-arm, open-label, multicentre study of darbepoetin alfa for treating anaemic patients with MDS.

**Fig 1:**
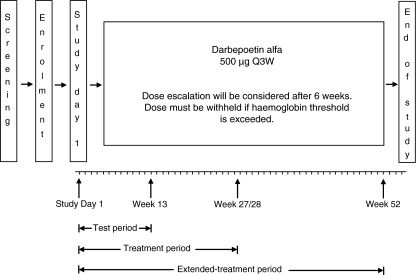
Study schema. Darbepoetin alfa was administered at 500 μg every 3 weeks (Q3W). After 6 weeks (at week 7), the dosing frequency could be escalated to every 2 weeks (Q2W). Treatment was withheld when a patient's haemoglobin reached ≥130 g/l. Darbepoetin alfa treatment was not to exceed 52 weeks, and end of study was planned for 3 weeks (at week 55 for Q3W dosing) or 2 weeks (at week 53 for Q2W dosing) after the last dose of darbepoetin alfa was administered. Study day 1 (the first day of darbepoetin alfa administration) to week 13 was designated as ‘test period’, study day 1 to week 27/28 was designated as ‘treatment period’ and study day 1 to week 52 was designated as ‘extended-treatment period’.

Study day 1 (no more than 4 d after enrolment) was the day on which the first dose of darbepoetin alfa was administered (Fig 1). This study was prospectively divided into specific time-points during treatment, which were related to the primary and secondary end-points of the study. These time-points included day 1 through to the end of week 13 (test period) and day 1 through to weeks 28 or 27 (treatment period, which varied depending on whether a patient was receiving study drug Q3W or Q2W. Continued treatment with darbepoetin alfa until week 52 (extended-treatment period) was also included in the study to allow for additional safety and biological assessments of the chronic administration of darbepoetin alfa in this setting (Fig 1).

Darbepoetin alfa treatment was initiated at a dose of 500 μg Q3W. All doses of darbepoetin alfa were administered subcutaneously. Darbepoetin alfa was escalated to 500 μg Q2W after 6 weeks (at week 7) of treatment for patients who did not achieve either ≥10 g/l haemoglobin increase over baseline levels or maintain haemoglobin levels in the range of 110–120 g/l. Subsequent doses were administered at the frequency received as of week 7, regardless of subsequent dose reductions. If haemoglobin concentrations increased to between 120 and 130 g/l, darbepoetin alfa was reduced to a dose of 300 μg at the same frequency as previously administered. For patients receiving 500 μg darbepoetin alfa and who had an increase >10 g/l in haemoglobin concentration in any 2-week period, the dose of study drug was reduced to 300 μg using the most recent dosing frequency. For patients who exceeded the haemoglobin threshold of ≥130 g/l, darbepoetin alfa was withheld until haemoglobin levels decreased to ≤120 g/l, wherein it was re-instated at 300 μg. For patients who did not maintain a haemoglobin concentration >110 g/l at reduced 300-μg darbepoetin alfa, doses (determined after 4 to 6 weeks at this dose) were escalated to 500 μg. Treatment was permanently discontinued for any patient who experienced a grade ≥3, non-haematological toxicity related to darbepoetin alfa treatment.

The end of study was planned for 2 or 3 weeks after the last dose of darbepoetin alfa was administered (i.e. week 55 for patients receiving study drug Q3W throughout the study and week 53 for patients who switched to Q2W dosing at the beginning of week 7).

### Study drug and concomitant medications

Darbepoetin alfa (Aranesp®) was supplied by Amgen Inc. (Thousand Oaks, CA, USA) as a clear, sterile protein solution of 500 μg darbepoetin alfa per ml of polysorbate-containing solution. Throughout the study, patients could be treated with any concomitant medications or treatments necessary to provide adequate supportive care except for those treatments listed in the exclusion criteria (see ‘Study population’). Iron supplementation was administered (to support erythropoiesis) according to the policy of each institution.

### Study end-points and assessments

The primary efficacy end-point was the percentage of patients who achieved either a major or minor erythroid response during the initial 13 weeks (test period) of darbepoetin alfa therapy. A major erythroid response was defined as either a ≥20 g/l increase in haemoglobin concentration from baseline (in the absence of a RBC transfusion within the preceding 28 d) or transfusion independence for patients who were transfusion-dependent at screening (transfusion dependence was defined as at least three RBC transfusions in the 3 months before screening). A minor erythroid response was defined as either an increase in haemoglobin concentration of ≥10 to <20 g/l from baseline (in the absence of a RBC transfusion within the preceding 28 d) or a 50% decrease in transfusion requirements for patients who were transfusion-dependent at screening. An alternative definition of erythroid response based on the updated IWG definition (Cheson *et al*, 2006) was used as a sensitivity analysis for responses during the extended treatment period by baseline eEPO categories. This was defined as an initial increase in haemoglobin of ≥15 g/l from baseline (in the absence of an RBC transfusion within the preceding 28 d) and an average increase in haemoglobin of ≥15 g/l from baseline that was sustained for 8 weeks following the initial rise. This definition made no distinction between major and minor responses.

Prospectively defined secondary end-points analysed during the extended treatment period of darbepoetin alfa therapy included the percentage of patients who achieved a major or minor erythroid response, incidence of haemoglobin response (haemoglobin concentration increase of ≥20 g/l from baseline in the absence of RBC transfusions within the preceding 28 d), the mean change in haemoglobin levels from baseline to week 53/55, the incidence of RBC transfusions and the mean change in the patient-reported Functional Assessment of Cancer Therapy-Fatigue (FACT-F) score from baseline to week 53/55. Additional end-points analysed during the extended treatment period included the incidence of achieving the targeted haemoglobin range of 110 to 130 g/l, the mean haemoglobin concentration maintained after achieving the haemoglobin target and the percentage of patients who maintained haemoglobin concentrations within the target range of 110–130 g/l. This haemoglobin target range was based on evidence-based guidelines to minimize transfusion requirements and to maximize quality of life (QOL) in cancer patients with anaemia (Rizzo *et al*, 2002; Bokemeyer *et al*, 2004; Rodgers *et al*, 2005). Additional QOL measures included the EQ-5D (Brooks *et al*, 2003) and three additional patient-related outcome questions concerning energy, activity and overall health. Safety end-points included incidence of adverse events, the percentage of patients who exceeded the haemoglobin threshold of ≥130 g/l, and the percentage of patients who withdrew from the study because of disease progression.

Complete blood count tests were performed weekly by a central laboratory from weeks 1 to 13 and then Q3W from weeks 13 to 28 (or Q2W from weeks 13 to 27 if the dose frequency was escalated at week 7). Haemoglobin concentrations were also measured at each dosing visit by a local laboratory to determine if any dose adjustments were necessary. A central laboratory was used to determine the presence of antibodies to darbepoetin alfa at baseline, at week 27/28 and at the end of study.

### Statistical analysis

This final analysis included all data up to week 53/55. Analyses were stratified by the patient's previous ESA therapy status (ESA-naïve vs. prior ESA-treated), as specified in the study protocol. sas (version 9, Cary, NC, USA) was used for all data analyses. Categorical variables were summarized using frequencies and percentages and continuous variables were summarized using means, standard deviations and 95% confidence limits (CLs).

The primary and the safety analysis set consisted of all enroled patients who received at least one dose of darbepoetin alfa. The patient-reported outcome (PRO) analysis set consisted of all enroled patients who received at least one dose of darbepoetin alfa and completed both the baseline and at least one subsequent FACT-F questionnaire.

Missing haemoglobin data, either because of a missed visit or collected within 28 d after a RBC transfusion was imputed in the last-value-carried-forward (LVCF) approach. In the available data approach, missing haemoglobin values were not imputed. In the summaries of PRO end-points, the available data approach was used, and the data were not adjusted for RBC transfusions.

The crude proportions of patients who had a major erythroid response or a minor erythroid response were summarized, and 95% CLs were constructed for the percentages of patients in each category by using the normal approximations. The percentages of patients who had a haemoglobin response, achieved the target haemoglobin range, or received at least one RBC transfusion were estimated using the Kaplan–Meier (KM) method (95% CLs were based on the normal approximation with the variance estimated using Greenwood's formula). Estimates of the median times to event (with 95% CLs) were calculated and KM plots of the time to haemoglobin response and time to target haemoglobin were generated.

All adverse events were summarized by system organ class and preferred term according to the Medical Dictionary for Regulatory Activities (MedDRA version 9).

## Results

### Study population

This study began accruing patients on 14 May 2004 and completed enrolment on 6 July 2005 with 80 centres enroling a total of 209 patients. As 3 patients never received darbepoetin alfa, analyses were conducted on a primary analysis set of 206 patients. Of the 206 patients, 18% had significant protocol deviations (14% failed to meet inclusion/exclusion criteria and 5% received other ESAs during this study).

Of these patients, 144 patients (70%) had never been treated with an ESA before enrolment (ESA-naïve) and 62 patients (30%) had been treated with an ESA before enrolment (prior ESA-treated) (Table I). Most patients in the ESA-naïve and prior ESA-treated strata were Caucasian and had a median age of about 76 years. The prior ESA-treated patients began their ESA treatment a mean (SD) of 609 (638) d prior to study enrolment (median, 398 d). Seventeen (27%) of these patients received darbepoetin alfa as their most recent ESA therapy, with a mean (SD) dose of 232 (53) μg. Most (59%) of those patients who had prior darbepoetin alfa treatment were receiving Q2W dosing at their last dose. Forty-five (73%) of the prior ESA-treated patients received Epoetin alfa as their most recent ESA therapy, with a mean (SD) dose of 52 326 (18 528) U. Sixty-nine per cent of prior Epoetin alfa-treated patients received weekly dosing at the last dose prior to study entry.

**Table I:** Demographics, baseline characteristics and disease state of patients.

	ESA-naïve	Prior ESA-treated
	*n* = 144	*n* = 62
Sex, *n* (%)		
Men	72 (50)	31 (50)
Race, *n* (%)		
Caucasian	124 (86)	51 (82)
Other	20 (14)	11 (18)
Age, years		
Mean (SD)	75·0 (10·0)	75·7 (9·3)
Median (Min, Max)	76·0 (51, 93)	76·5 (55, 94)
Baseline eEPO category, *n* (%)		
Less than 100 mU/ml	97 (67)	40 (65)
100 to less than 500 mU/ml	30 (21)	13 (21)
Greater than or equal to 500 mU/ml	14 (10)	7 (11)
Missing	3 (2)	2 (3)
FAB classification, *n* (%)		
RA	81 (56)	38 (61)
RARS	53 (37)	20 (32)
RAEB	10 (7)	3 (5)
Missing	0 (0)	1 (2)
IPSS classification, *n* (%)		
Low	94 (65)	44 (71)
Intermediate-1	44 (31)	14 (23)
Missing	6 (4)	4 (6)
ECOG status, *n* (%)		
0 to 1	130 (90)	55 (89)
2	10 (7)	3 (5)
Missing	4 (3)	4 (6)
Mean (SD) baseline haemoglobin, g/l	97 (10) (*n* = 126)	100 (11) (*n* = 49)
Absolute neutrophil count <1·5 × 10^9^/l, *n* (%)	22 (15)	8 (13)
Platelets <100 × 10^9^/l, *n* (%)	23 (16)	11 (18)
Cytogenetics, *n* (%)		
Good	115 (80)	48 (77)
Intermediate	14 (10)	6 (10)
Poor	6 (4)	3 (5)
Unknown or not done	9 (6)	5 (8)
Transfusion-dependent, *n* (%)*	2 (1)	7 (11)
Mean (95% CI) baseline FACT-F score	30·1 (27·7, 32·6) (*n* = 115)	31·1 (27·6, 34·7) (*n* = 42)

ESA, erythropoiesis-stimulating agent; Min, minimum; Max, maximum; eEPO, endogenous erythropoietin; FAB, French–American–British; RA, refractory anaemia; RARS, refractory anaemia with ringed sideroblasts; RAEB, refractory anaemia with excess blasts; IPSS, International Prognostic Scoring System; ECOG, Eastern Cooperative Oncology Group; FACT-F, Functional Assessment of Cancer Therapy-Fatigue.

*Patients who received at least three RBC transfusions in 3 months before screening were considered transfusion-dependent.

Approximately two-thirds of all patients had baseline eEPO concentrations of <100 mU/ml. Both ESA-naïve and prior ESA-treated patients had a similar distribution of baseline eEPO levels. The ESA-naïve patients had a mean (SD) haemoglobin concentration of 97 (10) g/l and the prior ESA-treated patients had a mean (SD) haemoglobin concentration of 100 (11) g/l prior to initiation of darbepoetin alfa. Sixty-one per cent of prior ESA-treated patients had an FAB classification of RA and 71% had an IPSS classification of low-risk MDS. In the ESA-naïve group, 56% had an FAB classification of RA and 65% had an IPSS classification of low-risk MDS. At screening, 2 (1%) of ESA-naïve patients and 7 (11%) of prior ESA-treated patients were considered to be transfusion-dependent per protocol (had received at least three RBC transfusions in the 3 months before screening).

The average mean (SD) weekly dose of darbepoetin alfa administered during the entire study was 151 μg/week (SD = 62) to ESA-naïve patients and 174 (58) μg/week to prior ESA-treated patients. ESA-naïve patients were dosed for a mean (SD = 16) of 41 weeks and prior ESA-treated patients for 33 (20) weeks. The mean (SD) total number of doses received was 14 (7) doses in the ESA-naïve group and 13 (8) doses in the prior ESA-treated group. The percentage of patients who had a dose withheld for reaching the haemoglobin concentration threshold (≥130 g/l) was 34% in the ESA-naïve group and 26% in the prior ESA-treated group. A total of 90 patients (44%) required a dose increase per protocol specifications from Q3W to Q2W; 39% of ESA-naïve patients and 55% of prior ESA-treated patients had this increase in dose frequency.

### Erythroid response at week 13

**Fig 2:**
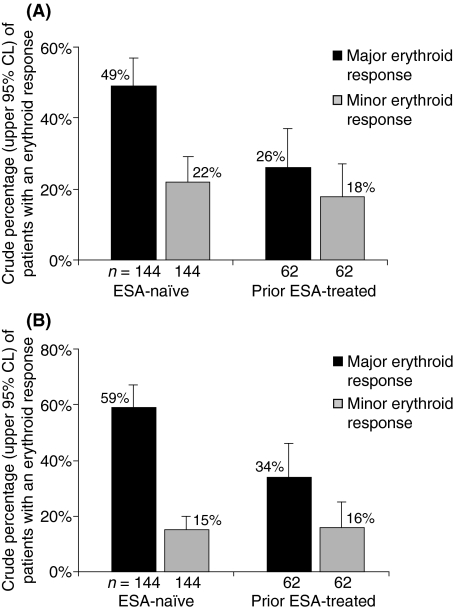
Patients who achieved an erythroid response by 13 and 53/55 weeks. A major erythroid response was defined as: i) an increase in Hb concentration of ≥20 g/l from baseline or ii) transfusion-independent for patients who were transfusion-dependent at screening. A minor erythroid response was defined as: i) an increase in Hb concentration of ≥10 and <20 g/l from baseline or ii) ≥50% decrease in transfusion requirements for patients who were transfusion-dependent at screening. (A) Percentage of patients achieving an erythroid response after 13 weeks of darbepoetin alfa therapy (the primary end-point). (B) The percentage of patients with an erythroid response after 53/55 weeks of darbepoetin alfa therapy. Error bars indicate the upper 95% CL. ESA, erythropoiesis-stimulating agent; CL, confidence limit.

The primary efficacy end-point in this study was the percentage of patients who achieved an erythroid response (either major or minor response) during the first 13 weeks of darbepoetin alfa therapy. In the ESA-naïve patients, the crude percentage (95% CL) that achieved a major erythroid response was 49% (40, 57), with a minor erythroid response rate of 22% (15, 29) (Fig 2A). In the prior ESA-treated patients, the crude percentage (95% CL) that achieved a major erythroid response was 26% (15, 37) and the crude percentage (95% CL) with a minor erythroid response was 18% (8, 27). A haemoglobin concentration increase of ≥20 g/l from baseline (which is also the definition of a haemoglobin response) was observed in all patients with a major erythroid response.

### Response outside the test period

Seventy-seven patients did not have a major or minor erythroid response in the 13-week test period (42 ESA-naïve and 35 prior ESA-treated). Among these 77 patients, 4 patients from each stratum (10% of ESA-naïve and 11% of prior ESA-treated) subsequently had an initial erythroid response during weeks 13 to 27/28 of the treatment period. No patients had an initial erythroid response during weeks 27/28 to 53/55 of the extended treatment period.

### Erythroid and haemoglobin responses at week 53/55

The incidence of erythroid responses after 53/55 weeks of treatment was also evaluated. In the ESA-naïve patients, the crude percentage (95% CL) that achieved a major erythroid response was 59% (51, 67), with a minor erythroid response rate of 15% (9, 20) (Fig 2B). In the prior ESA-treated patients, the crude percentage (95% CL) that achieved a major erythroid response was 34% (22, 46) and the crude percentage (95% CL) with a minor erythroid response was 16% (7, 25). Patients achieving a 20 g/l rise in haemoglobin concentration accounted for all major erythroid responses. Similar to responses observed during the 13-week test period, the percentage of patients who had a major response in the 53/55-week treatment period was almost twice as high in the ESA-naïve group as in the prior ESA-treated group. Among 119 patients who had a dose increase for any reason, 50 (42%) achieved a major erythroid response and an additional 20 (17%) achieved a minor erythroid response.

**Fig 3:**
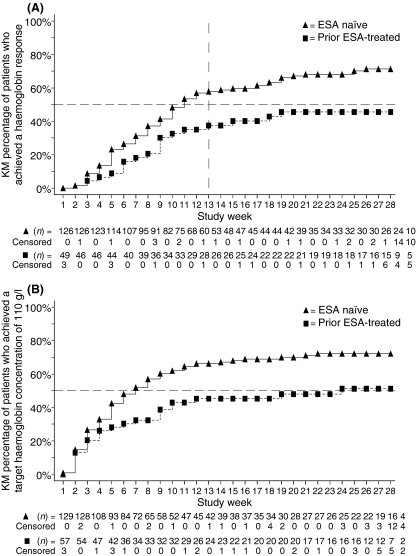
Kaplan–Meier plots of the time to haemoglobin response and of the time to target haemoglobin over the 28-week treatment period. (A) The time to haemoglobin response for erythropoiesis-stimulating agent (ESA)-naïve patients and prior ESA-treated patients. (B) The time to target haemoglobin for ESA-naïve patients and prior ESA-treated patients. *n* below each graph represents the number of patients at risk for an event (patients were censored after an end-point was achieved).

A separate analysis looked at the percentage of patients achieving a haemoglobin response (haemoglobin increase of ≥20 g/l from baseline in the absence of RBC transfusions within the preceding 28 d) after 53/55 weeks of darbepoetin alfa therapy. Thirty-one patients were not eligible for the haemoglobin response analysis as they had received RBC transfusions within 28 d of the baseline haemoglobin assessment, and thus had baseline haemoglobin values that were affected by recent transfusion. The KM percentage (95% CL) of eligible patients who achieved a haemoglobin response was 68% (60, 75). In ESA-naïve patients (*n* = 126), the KM percentage (95% CL) achieving a haemoglobin response was 73% (64, 81) and in prior ESA-treated patients (*n* = 49) the percentage of responders was 53% (37, 70). Figure 3A shows the KM curve for the time to haemoglobin response in both ESA-naïve and prior ESA-treated patients during the 28-week treatment period. As the haemoglobin response accounted for all patients with a major erythroid response, this figure also demonstrates the time to major erythroid response during the treatment period. The KM median (95% CL) time to haemoglobin response over the extended treatment period was 11 weeks (9, 15) in ESA-naïve patients and 31 weeks (13, NE) in prior ESA-treated patients (the upper 95% CL in prior ESA-treated patients could not be estimated).

The KM (95% CL) percentages of patients who attained the target haemoglobin range of 110 to 130 g/l and then maintained haemoglobin concentrations within the target range during the 53/55-week treatment period were also examined. Eighty-two per cent (70, 94) of ESA-naïve patients and 55% (40, 70) of prior ESA-treated patients achieved a target haemoglobin concentration of 110 g/l (Table II). Fig 3B shows the time to achieve the target haemoglobin range in both ESA-naïve and prior ESA-treated patient populations over the 28-week treatment period. Most ESA-naïve and prior ESA-treated patients who attained the target haemoglobin concentration were then able to maintain haemoglobin concentrations within the haemoglobin target range for the remainder of the study (Table II). After achieving the target haemoglobin concentration, no ESA-naïve patients and only two prior ESA-treated patients had their mean haemoglobin concentration drop to <100 g/l.

**Table II:** The percentage of patients who achieved the target haemoglobin concentration (110 g/l) and the mean haemoglobin concentration once target was achieved.

	ESA-naïve	Prior ESA-treated
	*n* = 144	*n* = 62
Patients eligible for target Hb analysis, n*	129	57
KM (95% CL) percentage who reached target Hb†	82% (70, 94)	55% (40, 70)
KM (95% CL) median time to achieve the target Hb, weeks	7 (5, 9)	24 (9, NE)
Patients eligible for mean Hb analysis after target achieved, *n*‡	105	31
Mean (SD) Hb concentration of maintenance, g/l	116 (8)	117 (8)
Median (Min, Max) Hb concentration of maintenance, g/l	117 (99, 133)	118 (101, 136)
Mean Hb category after achieving target, *n* (%)		
Less than 110 g/l	25 (24)	8 (26)
110–130 g/l	75 (71)	22 (71)
Greater than 130 g/l	5 (5)	1 (3)

ESA, erythropoiesis-stimulating agent; Hb, haemoglobin; KM, Kaplan–Meier; CL, confidence limits; Min, minimum; Max, maximum; NE, not estimable.

*Patients who had haemoglobin data either at baseline or after baseline were considered eligible except for those patients with baseline haemoglobin ≥110 g/l. Patients who had no haemoglobin data after baseline were counted as not having achieved the target level.

†Patients had to reach the target haemoglobin level (110 g/l) in the absence of a red blood cell transfusion within the preceding 28 d.

‡Patients who had baseline haemoglobin ≥110 g/l or who achieved the target haemoglobin concentration of 110 g/l were included.

**Fig 4:**
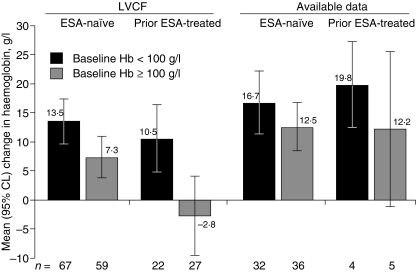
Change in haemoglobin from baseline to week 53/55. Data are presented from an analysis using the last-value-carried-forward (LVCF) approach and from an analysis using available data. The mean change in haemoglobin is shown by baseline haemoglobin category. Error bars indicate the upper and lower 95% CL. ESA, erythropoiesis-stimulating agent; CL, confidence limit.

The mean baseline haemoglobin concentrations for ESA-naïve and prior ESA-treated patients are presented in Table I. Using the LVCF approach to impute missing values, the mean (95% CL) change in haemoglobin concentration from baseline to week 53/55 was 11 g/l (8, 13) in ESA-naïve patients and 3 g/l (−2, 8) in prior ESA-treated patients (Fig 4). Using the available data approach, the mean (95% CL) change in haemoglobin to week 53/55 was 14 g/l (11, 18) for ESA-naïve patients (*n* = 68) and 16 g/l (9, 22) for prior ESA-treated patients (*n* = 9).

### Factors associated with erythroid response

#### IPSS score

**Fig 5:**
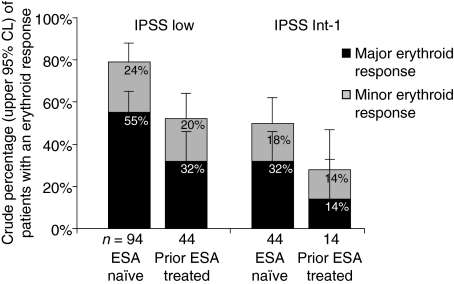
Major and minor erythroid responses during the 13-week test period by IPSS classification. The percentage of patients achieving an erythroid response during the 13-week test period is shown for patients with Low and Intermediate-1 (Int-1) IPSS classifications, and stratified by prior ESA treatment status. IPSS, International Prognostic Scoring System; ESA, erythropoiesis-stimulating agent; CL, confidence limit.

For patients classified as low risk according to the IPSS, the crude percentage (95% CL) that experienced major erythroid responses during the 13-week test period was 55% (45, 65) for ESA-naïve patients and 32% (18, 46) for prior ESA-treated patients. Patients classified in the Intermediate-1 IPSS risk class had lower rates of response [32% (18, 46) for ESA-naïve and 14% (0, 33) for prior ESA-treated] (Fig 5).

#### Baseline serum eEPO

Patients with lower serum eEPO measured at baseline appeared to have a higher rate of haematological response. Using the updated IWG definition for erythroid response that does not distinguish between major and minor responses (Cheson *et al*, 2006), ESA-naïve patients at week 53/55 had responses that ranged from 60% for those with baseline eEPO levels <100 mU/ml to 14% in those with baseline eEPO levels ≥500 mU/ml. In prior ESA-treated patients, erythroid responses were approximately 30% for both these baseline eEPO categories (Table III).

**Table III:** The percentage of patients who achieved an erythroid response over the 53/55-week study classified by baseline eEPO.

	ESA-naïve	Prior ESA-treated
Patients achieving an erythroid response, *n* (%)*		
Baseline eEPO <100 mU/ml	58 (60) (*n* = 97)	12 (30) (*n* = 40)
Baseline eEPO ≥100 mU/ml and <500 mU/ml	13 (43) (*n* = 30)	2 (15) (*n* = 13)
Baseline eEPO ≥500 mU/ml	2 (14) (*n* = 14)	2 (29) (*n* = 7)

ESA, erythropoiesis-stimulating agent; eEPO, endogenous erythropoietin.

*Erythroid response was defined as an initial increase in haemoglobin of ≥15 g/l from baseline (in the absence of an RBC transfusion within the preceding 28 d) and an average increase in haemoglobin of ≥15 g/l from baseline that was sustained for at least 8 weeks following the initial rise.

### Transfusions

During weeks 1 to 13 of the study, the KM percentage (95% CL) of patients with transfusions was 17% (11, 23) in the ESA-naïve group and 35% (22, 47) in the prior ESA-treated group. During weeks 1 to 28, the KM percentage (95% CL) of patients with transfusions was 18% (12, 25) in the ESA-naïve group and 37% (24, 50) in the prior ESA-treated group. Over the course of the study, the KM percentage (95% CL) of patients with RBC transfusions was 28% (20, 36) in the ESA-naïve group and 42% (29, 56) in the prior ESA-treated group.

Of the nine patients who were transfusion-dependent at study entry, 1 ESA-treated patient achieved transfusion independence for a period during the study. Based on a comparison of the weekly transfusion rate before screening and the transfusion rate between 28/29 and 53/55 weeks of treatment, 1 patient from each group achieved a ≥50% decrease in transfusions; the remainder of those who were transfusion-dependent at screening achieved a <50% decrease in transfusions (1 patient in the ESA-naïve group and 6 patients in the ESA-treated group).

### Change in QOL scores

**Fig 6:**
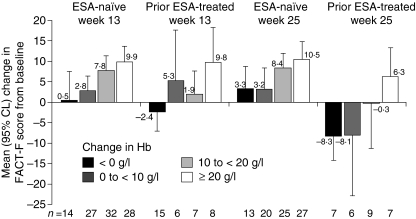
Change in Functional Assessment of Cancer Therapy-Fatigue (FACT-F) score from baseline. The mean change in the FACT-F score from baseline to weeks 13 and 25 is shown by mean changes in haemoglobin levels. Error bars indicate the upper or lower 95% CL. ESA, erythropoiesis-stimulating agent; CL, confidence limit; Hb, haemoglobin.

The baseline FACT-F scores for the ESA-naïve and prior ESA-treated patients are presented in Table I. The mean (95% CL) change in FACT-F score from baseline to week 13 was 5·6 (3·5, 7·7) points in the ESA-naïve patients (*n* = 104) and 2·4 (−0·9, 5·8) points in the prior ESA-treated patients (*n* = 36) (Fig 6). The mean (95% CL) change in FACT-F score from baseline to week 53/55 was 6·2 (4·0, 8·4) points in the ESA-naïve patients (*n* = 70) and 6·2 (1·7, 10·7) points in the prior ESA-treated patients (*n* = 17). Increases in FACT-F scores were associated with improvements in haemoglobin levels from baseline (Fig 6). An increase of ≥3 points in the FACT-F score has previously been reported to be clinically significant (Cella *et al*, 2002). A separate *ad hoc* analysis of FACT-F by FAB classification did not reveal meaningful information because of the unequal numbers of patients in each class. The EQ-5D and additional patient questionnaire items collected from 75% of enroled patients showed no trends over time.

### Safety end-points

Of the 206 patients, 92% reported an adverse event (both treatment-unrelated and related) during the 53/55-week treatment period [93% of ESA-naïve patients (*n* = 144) and 89% of prior ESA-treated patients (*n* = 62)]. The most common adverse events reported were fatigue (37%), peripheral oedema (18%), dyspnoea (17%) and dizziness (16%). Events occurring in a notably greater proportion of erythropoietin-treated patients than erythropoietin-naïve patients were fatigue (47% vs. 33%), anaemia (16% vs. 7%) and upper respiratory tract infection (15% vs. 4%). Events that occurred in a notably greater proportion of erythropoietin-naïve patients than erythropoietin-treated patients were dizziness (19% vs. 10%) and pain in extremity (13% vs. 5%). Twenty-eight per cent of ESA-naïve patients and 34% of prior ESA-treated patients experienced serious adverse events with the most common serious adverse event being infection (e.g., pneumonia or sepsis).

The incidence of treatment-related adverse events was low over the 53/55-week study period. Two patients (in the ESA-naïve group) experienced serious treatment-related adverse events. One patient experienced a moderate transient ischaemic attack at study week 37 and discontinued study participation in response to the event which resolved on the date of onset. The patient's haemoglobin level was determined to be 93 g/l at the time of the event. Another patient experienced severe hypertension at study week 10. After temporary discontinuation of darbepoetin alfa, the hypertension resolved 10 d after onset.

Thromboembolic events (including deep vein thrombosis or other thrombosis) were reported in 4 patients (2%). These included aortic stenosis requiring bypass surgery, retinal artery occlusion and thrombosis categorized as a mild blood clot on the inner forearm. One patient experienced coagulopathy with coumadin listed as the probable causative agent. All four thromboembolic events resolved and none were judged by the investigator to be related to study medication. An additional 24 patients experienced events that may have been related to embolism or thrombosis. Among patients who had a rapid rise in haemoglobin level (defined as >15 g/l in 21 d or >20 g/l in 28 d, adjusting for RBC transfusions), 12 patients (10%) experienced thromboembolic or related events compared with 16 patients (18%) without a rapid rise in haemoglobin. Of those patients who exceeded the haemoglobin threshold of 130 g/l, 6 patients (10%) experienced thromboembolic or related events compared with 22 patients (15%) who did not exceed the haemoglobin threshold. All but two of these events resolved, and only the aforementioned transient ischaemic attack was considered treatment-related. These additional cardiovascular events are included with other adverse events of interest as summarized in Table IV.

**Table IV:** Adverse events of interest.

	ESA-naïve	Prior ESA-treated
	*n* = 144	*n* = 62
Patients who had adverse events of interest, *n* (%)	34 (24)	21 (34)
Neoplasms benign, malignant and unspecified, *n* (%)	12 (8)	9 (15)
Hypertension, *n* (%)	7 (5)	4 (6)
Cardiovascular and thromboembolic events	18 (12)	10 (16)
Arrhythmias, *n* (%)	5 (3)	2 (3)
Cerebrovascular accident, *n* (%)*	6 (4)	1 (2)
Congestive heart failure, *n* (%)	4 (3)	2 (3)
Myocardial infarction/coronary artery disorders, *n* (%)	3 (2)	3 (5)
Embolism/thrombosis, *n* (%)	1 (1)	3 (5)
Immune system disorders, *n* (%)	1 (1)	0 (0)
Seizure, *n* (%)	1 (1)	0 (0)
Pure red cell aplasia	0 (0)	0 (0)

ESA, erythropoiesis-stimulating agent.

*Includes five thrombotic events, one haemorrhagic event and one event of unknown aetiology.

During the study, 10 patients (5%) reported disease progression. Six of these were progression to AML or other leukaemias as follows: 5 patients from the ESA-naïve group including two patients with RAEB classification (ages 64 and 76 years both discontinued study at week 29), 1 patient with RAEB classification (age 81 years, discontinued at week 49), 1 patient with RA classification (age 65 years, discontinued at week 30), 1 patient with RA classification (age 83 years, discontinued at week 19); and 1 patient from the prior ESA-treated group with RAEB classification (age 82 years, discontinued at week 1). Four additional patients (3 in the ESA-naïve group and 1 in the prior ESA-treated group) discontinued study at weeks 17, 46, 49 and 27 because of disease progression of unspecified type.

Eight ESA-naïve patients (6%) and 7 prior ESA-treated patients (11%) died during the study. The causes of death included pneumonia, sepsis, neoplasms (i.e. disease progression), cardiac disorders, respiratory arrest and unknown causes. With the exception of pneumonia occurring in 2 patients, no events leading to death occurred in more than one patient.

Of the 206 patients in the primary analysis set, a total of 115 patients (56%) completed the study. A higher percentage of erythropoietin-naïve patients (63%) than erythropoietin-treated patients (40%) completed the study. Among patients who did not complete the study, the most common reasons were administrative decision (12%), other (7%), consent withdrawn (7%), adverse event (6%) and death (5%). No patients had positive results in the darbepoetin alfa neutralizing antibody assay in this study.

## Discussion

Erythropoiesis-stimulating agent therapy may be able to correct anaemia in MDS patients by enhancing the survival and maturation of erythroid precursors (Hofmann & Koeffler, 2005). Previous studies have shown that recombinant human erythropoietin (rHuEPO) can induce a response (raise haemoglobin levels and reduce transfusion requirements) in about 30% of low-risk MDS patients (Rose *et al*, 1995; Hellstrom-Lindberg *et al*, 1998; Hellstrom-Lindberg, 2005). When administered with G-CSF, the percentage of low-risk MDS patients who respond to rHuEPO therapy may increase to about 40% (Casadevall *et al*, 2004; Catenacci & Schiller, 2005; Balleari *et al*, 2006), particularly in the RARS subtype (Hellstrom-Lindberg *et al*, 1998, 2003). Several evidence-based guidelines have supported the use of ESAs for treating anaemia in MDS patients, albeit at higher doses than those used for patients with CIA (Alessandrino *et al*, 2002; Greenberg *et al*, 2006).

This study suggests that darbepoetin alfa may also be an effective therapy for treating anaemia associated with MDS. Results indicated that darbepoetin alfa 500 μg Q3W was well tolerated and capable of meaningfully increasing haemoglobin levels over a 53/55-week period in low-risk MDS patients. The study patients were able to achieve a major erythroid response, a target haemoglobin of 110 g/l and maintain haemoglobin in the range of 110 to 130 g/l (consistent with the range recommended by evidence-based guidelines at the time for ESA therapy) (Rizzo *et al*, 2002; Bokemeyer *et al*, 2004; Rodgers *et al*, 2005). In addition, treatment with darbepoetin alfa appeared to reduce fatigue. Transfusion requirements were 28% in ESA-naïve and 42% in prior ESA-treated populations over the 53/55-week treatment period. An effect of darbepoetin alfa therapy on transfusion incidence over time may have been difficult to detect because overall transfusion requirements in this study were relatively low, reflecting the fact that most patients in this study were not transfusion-dependent at screening. As the study did not include an untreated control group, further assessment of efficacy was not possible.

It is important to identify patients for whom ESA treatment may have a lower probability of success. Although not an end-point of this study, based on previously suggested predictors of haematological response (Rose *et al*, 1995; Hellstrom-Lindberg *et al*, 2003; Musto *et al*, 2005), we examined factors associated with erythroid responses. In this study, most patients who achieved an erythroid response (major or minor) during the study did so during the 13-week test period. The low percentage of patients who had a first erythroid response during weeks 13 to 27/28 of the treatment period and the lack of any initial erythroid responses from week 27/28 to the end of study suggest that patients without a change in haemoglobin by 13 weeks of treatment independent of transfusion may not benefit from continued therapy. In patients with eEPO levels <100 mU/ml, a trend towards more favourable response was observed in accordance with previous reports (Rose *et al*, 1995; Hellstrom-Lindberg *et al*, 2003; Musto *et al*, 2005). Patients with a low risk score in the IPSS classification system also appeared to respond to ESA treatment more frequently than did those with an Intermediate-1 score (Fig 5). Baseline transfusion requirement is another recognized predictor of response to therapy. However, because of the low number of patients receiving frequent transfusions at study entry (Table I), this could not be used as a predictive factor in this study. It is interesting to note that patients who were ESA-naïve responded more robustly to treatment with darbepoetin alfa than patients who had been previously treated with an ESA. The reasons for the response difference by prior treatment status are unclear. As the majority of previously ESA-treated patients had been on treatment for over a year, there were probably differences in duration of disease between the groups. A further contributing factor may have been the lower baseline eEPO values in the ESA-naïve group (Table III).

The safety profile reported in this study was similar to that reported in other darbepoetin alfa studies in the setting of CIA (Vansteenkiste *et al*, 2002; Hedenus *et al*, 2003). During the 53/55-week treatment period, thromboembolic events only occurred in 2% of MDS patients, all of which resolved. This rate is somewhat lower than that observed in patients with CIA treated with ESAs. Overall, hypertension was experienced by 5% of patients, but occurred in 10% of patients who exceeded the threshold haemoglobin level of 130 g/l compared with 3% of those who remained below the haemoglobin threshold. This was the only adverse event that appeared potentially related to exceeding the haemoglobin threshold of 130 g/l in this study. This withholding level was consistent with the product information (Amgen Inc.) for darbepoetin alfa in CIA when this study was designed, although the current product information states that darbepoetin alfa be temporarily withheld if haemoglobin levels exceed 120 g/l in patients with CIA. We observed a low rate of disease progression to AML or other leukaemias, in accordance with other studies evaluating the use of darbepoetin alfa (Stasi *et al*, 2005; Mannone *et al*, 2006) or erythropoietin alfa (Hellstrom-Lindberg *et al*, 1998) in low-risk MDS patients. Two retrospective cohort studies have also shown no negative impact of ESAs on AML progression or overall survival in MDS patients (Jadersten *et al*, 2005, 2006).

Other studies have also examined the efficacy of darbepoetin alfa for treating anaemia in MDS patients. One pilot study and one phase 2 study have shown that darbepoetin alfa administered 150 μg/week to low- or intermediate-risk MDS patients can induce a major erythroid response in 35–40% of patients without apparent safety concerns (Musto *et al*, 2005; Stasi *et al*, 2005). In a phase 2 study that examined darbepoetin alfa administered 300 μg/week with or without G-CSF, 55% of low-risk MDS patients treated with darbepoetin alfa alone achieved a major erythroid response; additional responses were obtained in patients who had been unresponsive to darbepoetin alfa as a single agent when G-CSF was administered concomitantly with darbepoetin alfa (Mannone *et al*, 2006). Results from a recent retrospective cohort study suggested that darbepoetin alfa and Epoetin alfa were equally effective in treating MDS-induced anaemia (Patton *et al*, 2005).

In conclusion, the findings presented here suggest that treating low-risk MDS patients with darbepoetin alfa provides a clinical benefit for an otherwise debilitating chronic anaemia. This study of low-risk MDS patients treated with darbepoetin alfa for 53/55 weeks resulted in excellent response rates with infrequent drug dosing. The ability to administer darbepoetin alfa therapy at extended dosing intervals, such as Q2W or Q3W, may contribute to increased patient convenience by reducing the number of clinic visits for anaemia treatment. This feature of darbepoetin alfa may be especially advantageous for patients who require ESA therapy for treating anaemia over a long period of time. Future studies are needed to further investigate the efficacy and safety of darbepoetin alfa in MDS patients (both low-risk and high-risk) to identify predictors of response to darbepoetin alfa therapy and to also evaluate the impact of darbepoetin alfa therapy on QOL in this patient population.

## Appendix

In addition to the authors of this present paper, the following investigators and centres participated in this collaborative study:

Akhund, Birjis, Huntington Station, NY; Arena, Francis, Lake Success, NY; Armas, Armando, Boynton Beach, FL; Bari, Fazal, Mountain Lakes, NJ; Beeki, Venkatadri, Kansas City, MO; Behairy, Ahmed Soliman, Freesoil, MI; Bellam, Siva, Port Saint Lucie, FL; Bhatia, Andres, Gainesville, FL; Bhatt, Mukesh, Medina, OH; Boccia, Ralph, Bethesda, MD; Burke, John, Colorado Springs, CO; Carloss, Harry, Paducah, KY; Charu, Veena, Anaheim, CA; Chay, Christopher, Asheville, NC; Chowhan, Naveed, New Albany, IN; Davidson, Sheldon, Mission Hills, CA; Del Prete, Salvatore, Stamford, CT; DeLuca, Russell, Glen Burnie, MD; Diaz-Lacayo, Marvin, Aventura, FL; DiBella, Nicholas, Aurora, CO; Dold, Fred, Philadelphia, PA; Dreisbach, Luke, Rancho Mirage, CA; Elwood, Patrick, Columbus, OH; Esseesse, Isaac, Rockledge, FL; Gabrail, Nashat, Canton, OH; Gandhi, Sunhil, Lecanto, FL; Geils, Jr., George, Charleston, SC; Gersh, Robert, Spokane, WA; Hoffman, David, Beverly Hills, CA; Horvath, William, Lambertville, MI; Iannotti, Nicholas, Port Saint Lucie, FL; Irwin, David, Berkeley, CA; Kahanic, Stephen, Sioux City, IA; Kasubhai, Saifuddin, Tacoma, WA; Katakkar, Suresh, Tucson, AZ; Katzen, Harvey, Clinton, MD; Keaton, Mark, Augusta, GA; Knoll, Paul, Everett, WA; Kosmo, Michael, Poway, CA; McMahon, Richard, Littleton, CO; Mehta, Nilesh, Gurnee, IL; Noga, Stephen, Baltimore, MD; O’Rourke, Timothy, Grand Rapids, MI; Papish, Steven, Morristown, NJ; Patel, Ravi, Bakersfield, CA; Patel, Dhimant, Rhinelander, WI; Phatak, Pradyumna, Rochester, NY; Poth, James, Jackson, TN; Proothi, Subhash, Bethlehem, PA; Rangineni, Rajagopal, Saint Joseph, MO; Robbins, Gerald, New Port Richey, FL; Saba, Hussain, Tampa, FL; Saez, Ruben, Lakeland, FL; Saltzman, Marc, North Miami Beach, FL; Savin, Michael, Dallas, TX; Sawhney, Sumit, Tamarac, FL; Schergen, Alvin, Saint Louis, MO; Shao, Spencer, Portland, OR; Sjak-Shie, Nelida, Saint Louis, MO; Smith, David, Vancouver, WA; Smith, John, Portland, OR; Tchekmedyian, Nerses, Long Beach, CA; Vongkovit, Piyapong, Hickory, NC; Waterhouse, David, Cincinnati, OH; Wax, Michael, Cincinnati, OH; Willard, Ellen, Pinehurst, NC; Wood, Albert, Corpus Christi, TX; Zweibach, Alexander, New Braunfels, TX.
